# Decreasing severe pain and serious adverse events while moving intensive care unit patients: a prospective interventional study (the NURSE-DO project)

**DOI:** 10.1186/cc12683

**Published:** 2013-04-18

**Authors:** Audrey de Jong, Nicolas Molinari, Sylvie de Lattre, Claudine Gniadek, Julie Carr, Mathieu Conseil, Marie-Pierre Susbielles, Boris Jung, Samir Jaber, Gérald Chanques

**Affiliations:** 1 Intensive Care and Anesthesiology Department, University of Montpellier Saint Eloi Hospital, 80 Avenue Augustin Fliche, Montpellier, 34295, France; 2 Department of Statistics, University of Montpellier Lapeyronie Hospital, 39 Avenue Charles Flahaut, Montpellier, 34295, France; 3 Unité U1046 de l'Institut National de la Santé et de la Recherche Médicale (INSERM), Arnaud de Villeneuve Hospital, University of Montpellier 1, University of Montpellier 2, 39 Avenue Charles Flahaut, Montpellier, 34295, France

## Abstract

**Introduction:**

A quality-improvement project was conducted to reduce severe pain and stress-related events while moving ICU-patients.

**Methods:**

The Plan-Do-Check-Adjust cycle was studied during four one-month phases, separated by five-month interphases. All consecutive patients staying more than 24 hours were evaluated every morning while being moved for nursing care (bathing, massage, sheet-change, repositioning). Phase 1 was considered as the baseline. Implemented and adjusted quality-interventions were assessed at phases 2 and 3, respectively. An independent post-intervention control-audit was performed at Phase 4. Primary-endpoints were the incidence of severe pain defined by a behavioral pain scale > 5 or a 0 to 10 visual numeric rating scale > 6, and the incidence of serious adverse events (SAE): cardiac arrest, arrhythmias, tachycardia, bradycardia, hypertension, hypotension, desaturation, bradypnea or ventilatory distress. Pain, SAE, patients' characteristics and analgesia were compared among the phases by a multivariate mixed-effects model for repeated-measurements, adjusted on severity index, age, admission type (medical/surgical), intubation and sedation status.

**Results:**

During the four studied phases, 630 care procedures were analyzed in 53, 47, 43 and 50 patients, respectively. Incidence of severe pain decreased significantly from 16% (baseline) to 6% in Phase 3 (odds ratio (OR) = 0.33 (0.11; 0.98), *P *= 0.04) and 2% in Phase 4 (OR = 0.30 (0.12; 0.95), *P *= 0.02). Incidence of SAE decreased significantly from 37% (baseline) to 17% in Phase 3 and 21% in Phase 4. In multivariate analysis, SAE were independently associated with Phase 3 (OR = 0.40 (0.23; 0.72), *P *< 0.01), Phase 4 (OR = 0.53 (0.30; 0.92), *P *= 0.03), intubation status (OR = 1.91 (1.28; 2.85), *P *< 0.01) and severe pain (OR = 2.74 (1.54; 4.89), *P *< 0.001).

**Conclusions:**

Severe pain and serious adverse events are common and strongly associated while moving ICU patients for nursing procedures. Quality improvement of pain management is associated with a decrease of serious adverse events. Careful documentation of pain management during mobilization for nursing procedures could be implemented as a health quality indicator in the ICU.

## Introduction

Pain is a frequent event in intensive care unit (ICU) patients, with an incidence of moderate to severe pain during the ICU stay of up to 50% in medical as well as surgical patients [1-3]. Pain is associated with acute stress response including changes in heart rate, blood pressure, respiratory rate, neuro-endocrine secretion and psychological distress, such as agitation [4,5]. It has recently been reported that improved pain management was associated with improved patient outcome in the ICU [1,6-8]. However, pain remains currently under-evaluated and under-treated [3,9-12]. Therefore, pain management is highly challenging in the ICU setting.

One of the most common painful procedures in ICU patients is moving and turning for nursing care procedures (bathing, massage of back and pressure points, sheets change, repositioning) [3,13]. Pain during the first turning of the day is especially challenging to manage in our ICU. Indeed, this is often the longest turning time and includes the highest number of mobilizations and nursing care procedures. Moreover, the early morning nurses often have to manage ICU patients in collaboration with a reduced medical night-shift staffing, leading to necessarily greater nurse autonomy [14]. For instance, it has been reported for the past decade that between 50% of patients in the USA [9] and 80% in Europe [3,15] received no extra medication even though pain intensity increased during that procedure. More recently, a study assessing 330 turnings in 96 medical-surgical patients reported that the pain score significantly increased between rest and turning, while a bolus of analgesic was used in less than 15% of the turnings [16]. Moreover, serious adverse events (SAE) related to moving complex ICU patients are poorly documented. These SAE could be determined by the mobilization itself and/or the stress response associated with pain.

The present study was conducted to test the hypothesis that the implementation of a quality improvement process for pain management while moving ICU patients would be associated with a decreased incidence of both severe pain and SAE, and that those SAE would often be associated with pain events.

## Materials and methods

### Population

The study took place in the 16-bed medical-surgical ICU of St Eloi Hospital, a 660-bed teaching and referral facility of the University of Montpellier in France, staffed by 35 registered nurses (RNs), 25 nurse assistants, 3 certified registered nurse anesthetists, 7 attending physicians and 4 residents. Nurse to patient ratio was 1:2.5 as required in France [17]. The ICU has 24-hour anesthesiologist/intensivist medical staffing including three anesthesia residents and three attendings on dayshift, one resident and one attending on nightshift. RNs systematically and routinely assess pain and agitation at rest and during procedures using dedicated tools validated for ICU patients since 2003 [1]. For patients receiving a continuous infusion of sedatives, RNs have been using a sedation-analgesia algorithm since 2007 [18]. In the absence of continuous sedation, or previous analgesic ordering, a medical doctor was called in case of any pain or agitation events [1].

All consecutive patients ≥ 18 yrs old and staying in the ICU for more than 24 hrs were eligible. Exclusion criteria were decision to withdraw life-support within 48 hrs after admission and lacking data. Because of the observational, non-invasive design of this quality-improvement study based on the Plan-Do-Check-Adjust method which aimed to apply recommended practice guidelines [19], the need for written consent was waived as for previous published quality studies on sedation-analgesia practices in ICU patients [20] by the local scientific and ethics committee of Comité d'Organisation et de Gestion de l'Anesthésie Réanimation du Centre Hospitalier Universitaire de Montpellier (COGAR), which approved the conduct of the study.

### Study design

#### "Plan-step": multidisciplinary ICU work-group and choice of the studied procedure

**Figure 1:**
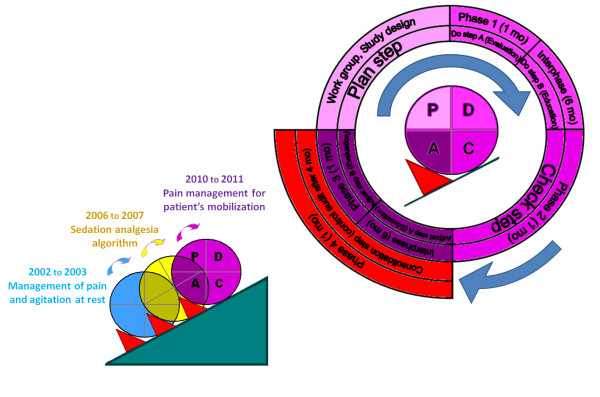
**Study-design and quality method**. This figure represents the quality-improvement process of pain and serious adverse events while moving ICU patients for turning and nursing care procedures. This 20-month process following the P-D-C-A steps was evaluated by four one-month studied phases separated by inter-study phases of four to six months. The present quality improvement process was the third quality process performed in the ICU regarding the management of sedation and analgesia. Consecutive improvement steps were followed according to the Plan-Do-Check-Adjust method for quality-improvement: - P (Plan-step): Multidisciplinary ICU work group creation, choice of the studied procedure and design of the quality improvement process. - D (Do-step): Beginning of the Nurse-Do study by a one-month baseline evaluation of pain management by nurse while moving the patients (studied Phase 1). Educational interventions for optimized pain management by nurse (Nurse-Do) started after the baseline studied phase. - C (Check step): One-month evaluation (Check) of educational interventions (studied Phase 2). - A (Adjust step): Adjustment of educational interventions implicating an increased multidisciplinary team collaboration, one-month evaluation (Check) of adjusted interventions (studied Phase 3). - Consolidation step: one-month control audit of the PDCA quality improvement process (studied Phase 4).

A multidisciplinary work-group was created, composed of three registered nurses, three assistant nurses, and three physicians (two attending physicians and one resident). All members received institutional education provided by the Hospital Pain Committee. Five meetings were necessary to elaborate the quality study design. The first nursing care procedure in the morning was chosen to be studied because it accounts in our ICU for the care which requires the longest duration of turning, including the largest number of moves and nursing care procedures in the day (bathing, massage of back and pressure points, sheet changing, repositioning, frequent change of dressings and placement of stockings and foot splints). Also, the work group had the impression that there was a strong contrast between the end and beginning of the day regarding pain, agitation and the number of alarms ringing from monitoring systems early in the morning. Contrary to pain at rest, pain during procedures was rarely reported in medical charts. We made the hypothesis that managing procedural pain during the first turning of the day would be the most challenging in our ICU. Figure 1 represents the study design that included four one-month studied phases separated by interphase periods of four to six months, according to the Plan-Do-Check-Adjust method [20-22]. Total length of the study was 20 months. The present quality improvement process was the third quality process performed in the ICU regarding the management of sedation and analgesia. The first quality improvement process, aimed at implementing a systematic assessment of pain and agitation in the ICU using validated tools, was initiated in 2002 and evaluated in 2003 [1]. The second project (2006 to 2007) was aimed at evaluating nurse interventions regarding a sedation-analgesia algorithm and at comparing them to a North American ICU [18].

#### "Do- step -A": studied phase-1 (February 2010)

Every first turning of the day, between 6 and 8 AM was evaluated (see below, evaluated parameters).

During this phase, a de-identified questionnaire was given to every RN and nurse assistant in order to assess their knowledge of written guidelines regarding sedation-analgesia in the ICU and their difficulties in managing sedation-analgesia routinely.

#### "Do-step B": first inter-study phase (March to August 2010)

Based on Phase 1 and questionnaire results, educational interventions were planned and educational posters were constructed and posted. Educational intervention was provided for all the nursing and medical staff by members of the work group during scheduled courses intended for 5 to 10 staff members at a time. Educational objectives included the ability: 1) to assess and control pain at rest before any moving procedure, 2) to determine the duration of peak analgesia for analgesic drugs used in the ICU in order to anticipate administration of drugs before moving a patient, 3) to escalate analgesic drugs according to the World Health Organization's analgesic steps in case of ineffectiveness after having referred to previous pain assessment, 4) to administer music therapy as well as other non-pharmacological analgesia therapies. Analgesics were selected by physicians according to the clinical situation and administered by RNs. Non-pharmacological therapies were selected and administered by the RN only. In order to develop the use of non-pharmacological therapies, headphones and dedicated pieces of music therapy were implemented in every patient's room. Music scores were composed by music therapists. The main characteristics of music (tempo, intensity, number of instruments) progressively decreased, then stabilized to a low pattern (slow tempo, low level of sound, one or two instruments), and finally increased slowly before removing the headphones from the patient. In other words, music characteristics followed a U-shape. Total duration of a music therapy session was 40 ± 5 minutes. Nurses and physicians were specifically educated by a music therapist during this interphase.

Finally, the clinical information system software was modified to include specificities of pain management for nursing care procedures. Posters referring to pain management and the sedation-analgesia algorithm were created to highlight educational objectives previously described. Posters were posted in every patient's room. These posters are shown in electronic supplement in their original French version as well as an English version (see Additional files 1, 2, 3, 4).

#### "Check-step ": studied Phase 2 (September 2010)

Every first turning of the day, between 6 and 8 AM was evaluated (see below, Evaluated parameters). This phase was aimed to measure the impact of the educational interventions.

#### "Adjust- step A": second inter-study phase (October 2010 to March 2011)

During six months (October 2010 to March 2011) a data and problems analysis was performed and multidisciplinary medical and nursing strategy was adjusted. As from this moment, medical staff was asked to systematically order one or more analgesics to be administered early in the morning before the nursing care procedures. Nurses had the possibility of using one or more of these analgesic drugs according to their discretion based on pain assessments. Moreover, pain management for the nursing procedure was standardized and systematically checked along with other nursing issues during daily medical rounds. Compliance with the quality improvement project was corrected by reminders and analysis of specific situations by the nurse manager and the ICU medical director during their weekly nursing medical round.

#### "Adjust-step B": studied Phase 3 (April 2011)

Every first turning of the day, between 6 and 8 AM was evaluated (see below, evaluated parameters). This phase was aimed to measure the impact of adjustments made during the second interphase.

#### "Consolidation- step": studied Phase 4 (September 2011)

A consolidation step was added to the PDCA-cycle to measure sustained quality-improvement as the new standard [21,23]. Therefore, a control-audit was realized by an independent observer, four months after the end of the study. All eligible patients were consecutively included. Choice of evaluated moving and nursing procedures was made by randomization using a random number generation method.

### Evaluated parameters

#### Studied phases 1, 2, 3

1) Pain was measured by the bedside RN while the patient was at rest before and during any moving procedures routinely throughout the study process, using validated ICU pain tools. Communicating patients rated their discomfort intensity on the visually enlarged numeric rating scale (NRS) from 0 (no pain) to 10 (maximum imaginable discomfort) [24]. For non-communicating patients (sedated or delirious patients), pain was assessed by nurses using the behavioral pain scale (BPS) for intubated patients [25] and the non-intubated BPS (BPS-NI) for non-intubated patients [26]. Severe pain events were defined by a NRS level > 6 according to the usual definition [27] or a BPS/BPS-NI score > 5 according to validation studies [25,26,28]. Those studies demonstrated a score > 5 for procedures known as very painful. Moderate pain was defined by a NRS level from 4 to 6 or a BPS > 3 (minimal score) but < 6. Awareness was assessed at baseline by the Richmond Agitation Sedation Scale (RASS) [29]. Inter-rater reliability of these sedation and pain scales has been assessed repeatedly in the ICU [1,18,26,30]. All bedside RNs present during the study phases were fully familiar with using these pain and sedation scales routinely, for both sedated and non-sedated patients.

2) SAE related to acute stress-response were assessed by physiological parameters (cardiac rhythm, heart rate, mean arterial pressure, respiratory rate and oximetry), measured continuously by the ICU monitor and recorded before and while the moving procedure by the bedside RN on a sheet dedicated to the study. SAE were defined as cardiac arrest, a new arrhythmia event and clinically relevant changes before and during the procedure defined as follows:

- Tachycardia: heart rate ≥ 110 beats/minute (b/min) if < 100 b/min before the procedure

- Bradycardia: heart rate ≤ 60 b/min if > 70 b/min before

- Hypertension: mean arterial pressure ≥ 110 mmHg if < 100 mmHg before

- Hypotension: mean arterial pressure ≤ 65 mmHg if > 70 mmHg before

- Desaturation: oxygen saturation ≤ 90% if > 92% before

- Bradypnea: respiratory rate ≤ 10 c/min if > 10 c/min before

- Ventilatory distress: severe ventilator asynchrony (nonstop coughing or impossible ventilation) in mechanically ventilated patients and/or tachypnea (respiratory rate ≥ 35 c/min if it was < 35 c/min)

3) Pharmacological therapies given within four hours prior to the moving procedure were reported by the bedside nurse on the patient flow sheet. Non-pharmacological therapies (explanation of the nursing care procedure, therapeutic massage, music, music therapy) performed to decrease pain while being moved were reported by the bedside nurse on a sheet dedicated to the study.

4) Demographic and medical data were prospectively recorded. Age, gender, type of admission (medical or surgical) and Simplified Acute Physiological Score (SAPS) II [31] were collected within 24 hrs after ICU admission. Medical admission was defined by the absence of surgical intervention within seven days prior to ICU admission.

#### Studied Phase 4 (control audit)

Pain was measured by bedside nurses at rest before and during any moving procedures, similarly to the other phases but reported routinely on the patient's flow sheet instead of a dedicated study sheet. Physiological parameters were recorded every 30 minutes by the patient's Clinical Information System (ICIP-Carevue, Philips-Medical-Systems, Eindhoven, The Netherlands). Maximal or minimal values, which had been recorded an hour before and after the moving procedure, were analyzed. Pharmacological therapies were evaluated as for the other phases. Non-pharmacological therapies were not assessed because of the absence of systematic notification in the medical chart.

### Endpoints

The primary endpoints were the incidence of severe pain defined by the proportion of patients who developed a severe pain event (BPS > 5 and/or NRS > 6) and the incidence of SAE defined by the proportion of patients who developed at least one SAE while being moved. Secondary endpoints were incidence of moderate pain, the existence of a relationship between pain and SAE, and a change in analgesic ordering practice patterns.

### Statistical analysis

Based on previous data [24], an incidence of severe pain of 26% was observed in our ICU during mobilization for nursing care procedures. To show a 50% reduction of severe pain, *n *= 100 procedures needed to be analyzed for every phase, with alpha 0.05 and beta 0.10. Missing data were expected because bedside RNs would sometimes forget or not have enough time to fill in the sheets dedicated for the study due to an eventual high workload in the ICU. Taking into account missing data and the rate of empty rooms in the ICU at a given time, this meant enrolling consecutive patients hospitalized in the ICU within one month for every phase. Also, repeated one-month phases could allow for implementing the study effect (Hawthorne effect) into a routine process which was part of the quality-improvement project [32]. Reference (baseline) phase was Phase 1.

Quantitative data were shown as mean and standard deviation or median and 25^th ^to 75^th ^percentiles according to data distribution. Student *t-*test or Wilcoxon test (quantitative data) and chi-square test (qualitative data) were used to compare patients included in the four phases. Because moving for nursing care procedures was evaluated every day of the ICU stay, one patient could be evaluated several times (repeated measures).

Thus, pain events, serious adverse events and analgesic ordering were compared in univariate analysis using a generalized linear mixed-effects model for repeated measures, taking into account repeated measures as random variables. Multivariate analysis of pain events and SAE was secondly performed using a generalized linear mixed-effects model for repeated measures. Variables were selected if *P*-value was less than 0.20 in the univariate analysis and a stepwise procedure was used to select the final model. Furthermore, a sensitivity analysis was performed, removing tachycardia and hypertension, which are common events associated with pain, from the definition of SAE. This was done to measure the impact of the quality project on the incidence of other SAE. A *P*-value of ≤ .05 was considered statistically significant. Data were analyzed by a senior statistician from the Department of Statistics of the University of Montpellier Hospital using the R.2.13.0 software.

## Results

### Results from the questionnaire regarding sedation/analgesia practices

Among the nursing staff, 21 (60%) RNs and 17 (68%) nurse assistants answered the questionnaire during Phase 1. Pain assessment tools were thought to be adapted to ICU patients by all 21 (100%) RNs. Before the study, 17 (71%) RNs had already experienced a disagreement with doctors regarding pain management and 5 (29%) nurse assistants had experienced a disagreement with RNs. Disagreements occurred because some patients could have been in pain but physicians or nurses did not allow for increasing analgesics because of the risk of developing side-effects. Fourteen (58%) RNs did not refer to patients' previous pain assessments and analgesia documentation to better adjust analgesia for nursing care procedures for a given patient. Among the 21 RNs, 9 (43%) desired more autonomy in pain management. A greater autonomy was achieved in the quality improvement project by allowing nurses to administer selected analgesics. Because almost half of the nurses did not want greater autonomy, analgesic choice remained the physicians' role and pain management was developed more collaboratively between nurses and physicians. Educational interventions aimed at decreasing the incidence of severe pain and SAE and improving analgesics ordering were evaluated during the four studied phases.

### Evaluation of the quality improvement project across the four studied phases

**Figure 2:**
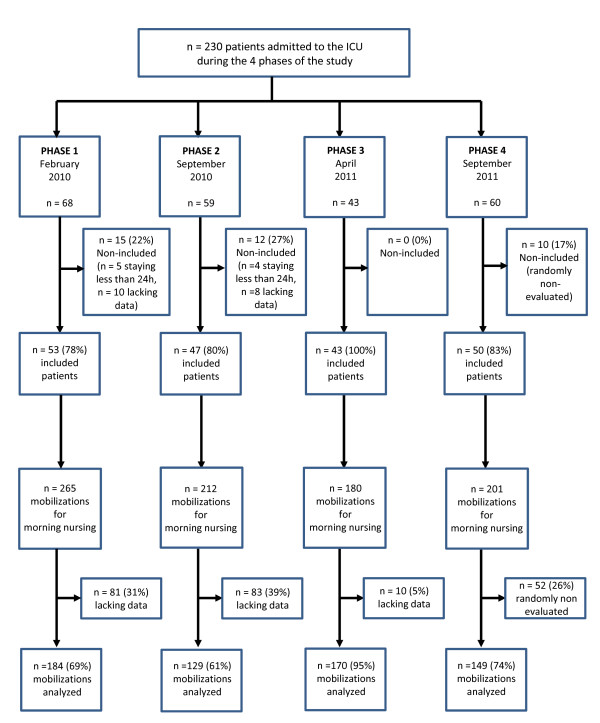
**Flow chart of the study**.

Overall 630 procedures were analyzed in 193 patients during the four studied phases, in 53, 47, 43 and 50 patients, respectively. The flow chart of the study is shown in Figure 2. Table 1 summarizes patients' demographic and medical characteristics. No significant difference was shown across groups except in Phase 3 during which patients had a significantly lower rate of procedures evaluated while receiving a continuous infusion of sedatives (propofol or midazolam).

**Table 1:** Characteristics of patients included in the four phases of the study.

	Phase 1 (baseline) *n *= 53	Phase 2 (intervention) *n *= 47	Phase 3 (adjustment) *n *= 43	Phase 4 (consolidation) *n *= 50	*P* 1-2	*P* 1-3	*P* 1-4
Age (years), median (IQR)	64 (54; 74)	65 (49; 74)	61 (49; 67)	61 (51;69)	0.87	0.10	0.24
Female Sex, n (%)	19 (36%)	18 (38%)	12 (28%)	13 (26%)	0.84	0.51	0.30
SAPS II, median (IQR)	41 (31; 54)	38 (27; 53)	34 (27; 41)	37 (26; 53)	0.64	0.07	0.57
Surgical admission*, n (%)	22 (42%)	25 (53%)	16 (37%)	26 (52%)	0.32	0.29	0.33
Mechanical ventilation, n (%)	24 (45%)	21 (45%)	13 (30%)	22 (45%)	0.95	0.13	0.90
Sustained use of sedatives, n (%)	22 (42%)	15 (32%)	9 (21%)	14 (29%)	0.32	0.03	0.15
RASS level, median (IQR)	0 (-3; 0)	0 (-1; 0)	0 (0; 0)	0 (-1; 0)	0.92	0.52	0.41
Number of procedures evaluated per patient, median (IQR)	2 (1; 4)	2 (1; 3)	3 (1; 5)	3 (3; 3)	0.39	0.38	0.24

IQR, Inter-Quartile-Range (25^th ^to 75^th ^percentiles); RASS, Richmond-Agitation-Sedation-Scale [29] from -5 (deep sedation) to +4 (combative agitation), a level of 0 defines an awake state of awareness without any agitation; SAPS II, Simplified-Acute-Physiology-Score II [31]. * Surgical patients all underwent abdominal surgery.

**Figure 3:**
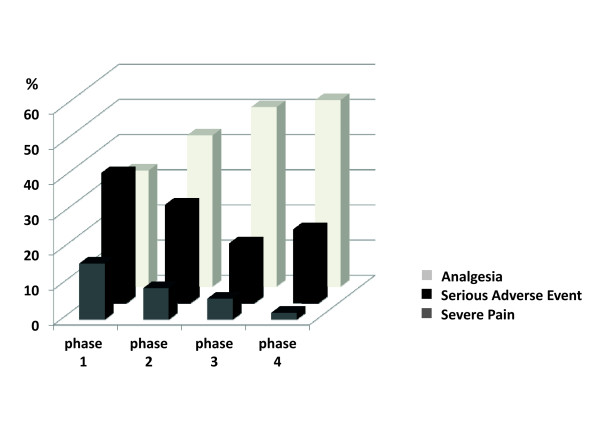
**Incidence of severe pain, serious adverse events and analgesia**. This figure shows that the incidence of severe pain and serious adverse events (SAE) decreased across the quality improvement study while the proportion of given analgesia increased. The difference was significant for severe pain (*P *= 0.04 and 0.02), SAE (*P *< 0.001 and *P *< 0.01) and analgesia (*P *= 0.01 and *P *< 0.01) between Phase 1 (baseline) and Phases 3 and 4, respectively.

Incidence of severe pain, as well as at least one SAE (cardiac arrest, arrhythmias, tachycardia, bradycardia, hypertension, hypotension, desaturation, bradypnea or ventilatory distress), decreased over the quality improvement study, while the proportion of analgesia given for nursing care procedures increased (Figure 3). The difference was not significant between Phase 1 (baseline) and Phase 2 (first intervention P-D-C-A step) but became significant during Phase 3 (adjusted-intervention P-D-C-A step) and Phase 4 (consolidation P-D-C-A step).

In multivariate analysis adjusted for cofactors and repeated measures (Table 2), severe pain was significantly less frequent during both Phase 3 (odds ratio (OR) = 0.33 (0.11; 0.98), *P *= 0.04) and Phase 4 (OR = 0.30 (0.12; 0.95), *P *= 0.02). Incidence of moderate pain did not significantly decrease during the study (see Additional file 5, Table S1).

**Table 2:** Factors associated with severe-pain determined by univariate and multivariate mixed-effects model analysis

	Univariate analysis				Multivariate analysis	
	
	All procedures (*n *= 632)	Severe pain (*n *= 61)	Others (*n *= 571)	*P*	OR (95%CI)	*P*
Phase 1, n (%)	184	30 (49%)	154 (27%)			
Phase 2, n (%)	129	12 (20%)	117 (20%)	0.22		
Phase 3, n (%)	170	11 (18%)	159 (28%)	0.04	0.33 (0.11; 0.98)	0.04
Phase 4, n (%)	149	8 (13%)	141 (25%)	0.03	0.30 (0.12; 0.95)	0.02
						
Age, median (IQR)	63 (51; 71)	64 (57; 76)	63 (51; 71)	0.16		
Female gender, n (%)	186 (29%)	17 (28%)	169 (30%)	0.94		
SAPS II, median (IQR)	39 (29;41)	39 (27;51)	39 (30;51)	0.48		
Surgical admission, n (%)	219 (35%)	25 (41%)	194 (34%)	0.23		
Intubation status, n (%)	216 (34%)	24 (39%)	192 (34%)	0.95		
Sustained use of sedatives, n (%)	114 (18%)	10 (16%)	104 (18%)	0.69		
RASS level, median (IQR)	0 (-1; 0)	0 (-1; 0)	0 (-1; 0)	0.31		

CI, Confidence-Interval; IQR, Inter-Quartile-Range (25^th ^to 75^th ^percentiles); OR, Odd-Ratio; RASS, Richmond-Agitation-Sedation-Scale [29]; SAPS II, Simplified-Acute-Physiology-Score II [31]. In addition to studied phases, variables were selected in multivariate analysis if *P*-value was less than 0.20 in the univariate analysis, that is, were included in the final mixed-effect model: studied phases and age.

A lower incidence of SAE was independently associated with Phase 3 (OR = 0.40 (0.23; 0.72), *P *< 0.01) and Phase 4 (OR = 0.53 (0.30; 0.92), *P *= 0.03) whereas a higher incidence of SAE was associated with intubated status (OR = 1.91 (1.28; 2.85), *P *< 0.01) and severe-pain (OR = 2.74 (1.54; 4.89), *P *< 0.001) (Table 3). Incidence of SAE was not associated with moderate pain. Detailed incidence of SAE is shown in Table 4. The sensitivity analysis showed that the incidence of at least one SAE (not taking into account tachycardia and/or hypertension) was also associated with Phase 3, Phase 4, intubation status and severe-pain (see Additional file 5, Table S2). Finally, hypotension was a little more frequent during Phase 4 but there was no significant association among hypotension, studied phases and analgesia (*P *= 0.60, mixed-effect model).

**Table 3:** Factors associated with serious adverse events determined by univariate and multivariate mixed-effects model analysis

	Univariate analysis				Multivariate analysis	
	
	All procedures (*n *= 632)	Serious adverse events YES (*n *= 164)	Serious adverse events NO (*n *= 468)	*P*	OR (95% CI)	*P*
Phase 1, n (%)	184 (29%)	68 (41%)	116 (25%)			
Phase 2, n (%)	129 (20%)	36 (22%)	93 (20%)	0.09		
Phase 3, n (%)	170 (27%)	29 (18%)	141 (30%)	< 0.001	0.40 (0.23; 0.72)	< 0.01
Phase 4, n (%)	149 (24%)	31 (19%)	118 (25%)	< 0.01	0.53 (0.30; 0.92)	0.03
						
Age, median (IQR)	63 (51; 71)	64 (56; 75)	62 (51; 70)	0.09		
Female gender, n (%)	186 (29%)	44 (27%)	142 (30%)	0.44		
SAPS II, median (IQR)	39 (29; 41)	39 (31; 53)	38 (28; 50)	0.19		
Surgical admission, n (%)	219 (35%)	104 (63%)	309 (66%)	0.54		
Intubation status, n (%)	216 (34%)	79 (48%)	137 (29%)	< 0.01	1.91 (1.28; 2.85)	< 0.01
Sustained use of sedatives, n (%)	114 (18%)	38 (23%)	76 (16%)	0.17		
RASS level, median (IQR)	0 (-1; 0)	0 (-2; 0)	0 (-1; 0)	0.05		
						
Pain during moving						
Moderate pain, n (%)	160 (25%)	41 (25%)	119 (25%)	0.81		
Severe pain, n (%)	61 (10%)	30 (18%)	31 (7%)	< 0.001	2.74 (1.54; 4.89)	< 0.001

CI, Confidence interval; IQR, Interquartile Range (25^th ^to 75^th ^percentiles); OR, Odds ratio; RASS, Richmond Agitation-Sedation Scale [29]; SAPS II, Simplified Acute Physiology Score II [31]. In addition to studied phases, variables were selected in multivariate-analysis if *P*-value was less than 0.20 in the univariate analysis, that is, were included in the final mixed-effect model: studied phases, age, SAPS II, intubation status, sustained use of sedatives, RASS level and severe-pain events.

**Table 4:** Incidence of serious adverse events during each phase of the study

	Phase 1 (baseline) *n *= 182	Phase 2 (intervention) *n *= 129	Phase 3 (adjustment) *n *= 170	Phase 4 (consolidation) *n *= 149	*P* 1-2	*P* 1-3	*P* 1-4
Cardiac arrest, n (%)	2 (1%)	0 (0%)	0 (0%)	0 (0%)			
Arrhythmias, n (%)	3 (2%)	4 (3%)	1 (1%)	0 (0%)			
Tachycardia, n (%)	5 (3%)	5 (4%)	3 (2%)	6 (4%)			
Bradycardia, n (%)	0 (0%)	2 (2%)	0 (0%)	0 (0%)			
Hypertension, n (%)	14 (8%)	2 (2%)	6 (4%)	11 (7%)			
Hypotension, n (%)	8 (4%)	5 (4%)	2 (1%)	9 (6%)			
Oxygen desaturation, n (%)	19 (10%)	15 (12%)	8 (5%)	6 (4%)			
Bradypnea, n (%)	2 (1%)	6 (5%)	0 (0%)	0 (0%)			
Ventilatory distress, n (%)	24 (13%)	13 (10%)	15 (9%)	2 (1%)			
							
At least one event, n (%)	68 (37%)	36 (28%)	29 (17%)	31 (21%)	0.09	< 0.001	0.005

Statistical analysis was performed using a generalized linear mixed-effects model for repeated measures.

There was a change in analgesic ordering practice patterns across the quality improvement project (Table 5). Use of tramadol was significantly higher in Phase 3 and in Phase 4 than in Phase 1. Administration of at least one analgesic drug was significantly higher in Phase 3 and in Phase 4. New non-pharmacological therapies were implemented in the study, such as music-therapy, which was displayed in each patient's room with dedicated headphones and music scores specifically composed for relaxation. However, if music therapy and the total number of non-pharmacological therapies used to treat pain significantly increased between Phase 1 and Phase 2, this increase was not sustained afterward (Table 5).

**Table 5:** Proportion of pharmacological and non-pharmacological therapies used during each phase of the study

	Phase 1 (baseline) *n *= 184	Phase 2 (intervention) *n *= 129	Phase 3 (adjustment) *n *= 170	Phase 4 (consolidation) *n *= 149	*P* 1-2	***P*** 1-3	***P*** 1-4
Analgesics drugs, n (%)							
WHO step 3	28 (15%)	32 (25%)	36 (21%)	33 (22%)	0.11	0.14	0.12
WHO step 2: tramadol	17 (9%)	25 (19%)	48 (28%)	49 (33%)	0.19	0.001	< 0.001
WHO step 1: acetaminophen	29 (16%)	23 (18%)	44 (26%)	36 (24%)	0.72	0.23	0.17
nefopam	22 (12%)	9 (7%)	26 (15%)	33 (22%)	0.24	0.71	0.11
At least one drug	60 (33%)	56 (43%)	86 (51%)	79 (53%)	0.22	0.01	0.002
Number of drugs per patient, mean (SD)	0.52 (0.70)	0.69 (0.77)	0.91(0.85)	1.01 (0.97)	0.30	0.008	< 0.001
							
Non pharmacological therapies							
Explication*, n (%)	158 (87%)	91(71%)	140 (82%)		0.01	0.62	ND
Massage, n (%)	120 (66%)	82 (64%)	64 (38%)		0.86	< 0.001	ND
Standard music listening, n (%)	12 (7%)	10 (8%)	4 (2%)		0.08	< 0.001	ND
Music therapy, n (%)	0 (0%)	5 (4%)	0 (0%)		0.99	1.00	ND
At least one therapy, n (%)	160 (88%)	98 (76%)	142 (84%)		0.06	0.54	ND
Number of therapies per patient, mean (SD)	2 (1;3]	3 (1;4]	1 (1;2]		< 0.01	0.05	ND

ND, not done (external audit of medical chart records); SD, Standard Deviation; WHO, World Health Organization. Analgesics were classified according to the WHO's pain relief ladder [55] used to treat pain. 1^st ^step, non-opioid analgesics; 2^nd ^step, minor opioids; 3^rd ^step, major opioids. Non-pharmacological therapies were evaluated for the three first phases. Non-pharmacological therapies were not assessed during the post-intervention Phase 4 (see text). * Explication of the procedure process, insurance that pain will be taken into consideration, if any.

## Discussion

The main findings of this quality improvement project are that moving an ICU patient for nursing care procedures is associated with severe adverse events (SAE) in one out of three procedures. The incidence of at least one SAE (cardiac arrest, arrhythmias, tachycardia, bradycardia, hypertension, hypotension, desaturation, bradypnea or ventilatory distress) is strongly associated with severe pain in multivariate analysis. A healthcare quality improvement project of pain management, while moving ICU patients, is associated with a decrease in both severe pain and SAE.

Being moved for nursing care procedures is one of the most painful procedures experienced by the patient during the ICU stay, whatever the type of admission (medical, surgical or trauma) [3,13,16,33]. Nevertheless, except for trauma and surgical patients, moving is currently not considered a painful procedure by ICU healthcare workers and physicians [34]. Similarly, to our knowledge, no study has reported yet whether pain might be a barrier for active mobilization in ICU patients and if a specific analgesia given to decrease pain while moving ICU patients would be associated with a greater chance to achieve rehabilitation objectives in the ICU setting [35,36].

One of the reasons not to treat pain is that ICU physicians may be uncomfortable ordering analgesic drugs [37] because of frequent organ dysfunction, altered pharmacokinetics and pharmacodynamics, and impaired mental status in critically ill patients [38]. Indeed, adverse events have been reported in critically ill patients even with non-opioid WHO's step-1 analgesics, such as acetaminophen [39] and nefopam [40]. In the present study, analgesics were administered upon nurse discretion but were chosen among eligible analgesics ordered by physicians according to the context and for each patient. Decreased incidence of severe pain and increased rate of analgesic administration observed during adjusted and consolidated steps of the quality project suggests that collaboration between nurses and physicians, which was the aim of educational intervention at the adjusted step, improved regarding appreciation of patients' pain and analgesics needs. A multidisciplinary discussion involving nurses and physicians/pharmacists is recommended regarding the complex management of pain in ICU patients [41]. To better define a rational plan for a given patient, it is important for physicians to assess nursing issues as it should be important for nurses to understand the benefit and risks associated with every analgesic ordered by physicians.

Tramadol was the only drug that's use significantly increased through the study. Except in the case of severe renal impairment, tramadol is an opioid associated with a minimal risk of ventilatory depression [42]. This could explain the preference of the team regarding its use in ICU patients who are at high risk of ventilatory depression. Similarly, tramadol use significantly increased in a previous quality improvement project aimed at reducing pain at rest in ICU patients [1]. In that study, incidence of pain significantly decreased through the quality improvement project as well as the duration of mechanical ventilation [1]. Similarly, in the present study, incidence of severe pain decreased as analgesic drug use increased without any increase of SAE. That could be attributed to an accurate evaluation of the benefit:risk ratio associated with analgesic ordering. Also, the incidence of moderate pain did not significantly decrease throughout the study. Actually, complete suppression of pain could be difficult or impossible in ICU patients considering the pain syndromes (surgery, trauma, acute pancreatitis) or contraindication of analgesic drugs in critical-illness (acetaminophen and liver dysfunction, anti-inflammatory drugs and renal dysfunction). In this way, American guidelines recommended defining an acceptable threshold of pain according to the context for each patient [43].

In order to reduce the risk of such drug adverse events, non-pharmacological therapies were developed throughout the study. Despite the implementation of music therapy as a new technology available for every patient and despite specific educational interventions, there was an increased use of non-pharmacological therapy but this increase was not sustained during the third phase of the study. Music therapy was poorly implemented throughout the project. Some nurses reported that the time which was required for a music therapy session (40 minutes) did not allow for easily preventing or treating procedural pain contrary to analgesic drugs. Also, nurses should have been more comfortable with analgesic drug use as the quality-improvement project was developed and might have discarded non-pharmacological therapies at the same time for different reasons including trust in their efficacy, timing and so on. If positive effects of music therapy and standard music listening have been shown in small-sized physiological studies in critically ill patients [44,45], the feasibility and impact of larger routine implementation has yet to be evaluated. Moreover, obstacles to widespread use of non-pharmacological therapy rather than analgesic drugs need to be explored because the rationale for development of non-pharmacological therapy in critical care is strong.

Decreased pain-associated stress response could partly explain the decrease of SAE observed during the last two studied phases (adjusted intervention and consolidation P-D-C-A-steps). Pain induces reflex responses that may alter respiratory mechanics and increase cardiac demand via tachycardia and increased myocardial oxygen consumption, leading to desaturation and blood pressure changes. Stress response may also induce hypercoagulability, immunosuppression and persistent catabolism [4,5]. In the present study, multivariate analysis adjusted to cofactors, such as severity of illness showed that severe pain events were an independent risk factor of SAE. This was confirmed by the sensitivity analysis, removing the most common pain-related adverse events (tachycardia and hypertension). Use of analgesics may decrease stress response in critically ill patients [46,47]. In our study, the main SAE observed were oxygen desaturation and ventilator distress (Table 4). The rate of these SAE decreased throughout the study, although ventilator management or oxygenation practices were not changed, contrary to pain management practices.

This study constitutes an improvement in quality and safety in healthcare. Such processes are fundamental to improving our healthcare, by changing our systems, avoiding overuse of ineffective care and underuse of effective care [48]. Quality improvement methods, such as the Plan-Do-Check-Adjust cycle, seek to apply proven treatments and recommended strategies to "real world" patients, allowing the integration of "best evidence" and "clinical evidence" [20,22]. To our knowledge, there are no published data regarding the feasibility of a quality improvement process for moving ICU patients. Changing practices is challenging in an ICU setting, with necessary education of a large team [49,50]. Moreover, a multidisciplinary approach is essential, placing responsibility with the team rather than with individuals. Differences in pain appreciation among physicians, nurses and assistant nurses are well known in the ICU setting [34] and were found again in our questionnaire. It has been previously reported that ICU physicians under-evaluated patients' pain compared to nurses [51], and that ICU nurses under-evaluated patients' pain compared to assistant nurses [52].

Our study has several limitations. First, there were less missing data in the third phase (adjusted intervention P-D-C-A phase) than in the two first phases. This could be explained by a high workload during February and September 2010, much higher than in April 2011. Indeed, one-third of the unit had to be closed unexpectedly in April after Phase 3 had begun. To deal with missing data and to avoid a possible bias due to more frequently evaluating patients in pain in the two first phases, patients were randomly enrolled in Phase 4. This phase (consolidation of P-D-C-A-steps) was aimed to reinforce the results observed in the previous phase [21,23]. Second, pain was evaluated by the bedside RN (BPS) or by the patient with the help of the bedside RN (NRS), and not by an independent investigator. However, this design is appropriate in a quality improvement process of routine care because self-evaluation of the caregiver is part of the improvement process [22,53]. Moreover, even if it was not possible to have an independent investigator at the bedside for all 16 patients during the turning every morning, the presence of an observer could have introduced another bias leading to more accurate care [32]. In this way, the study sheets were de-identified regarding the RN to allow for more independent evaluation of care. Also, the study design requires including all consecutive turnings within one month to deal with a possible punctual Hawthorne effect and to transform it in an acquired routine process [32]. The findings of this quality study can be supported by the incidence of SAE, which were objectively evaluated and also decreased along with the incidence of severe pain through the study. Third, if the global impact of educational interventions was supported by a decreased incidence of pain and SAE along with an increased rate of analgesic administration, no qualitative method was performed to better assess the impact of each aspect of educational interventions on health caregivers' skill regarding pain management as well as nurse-physician interaction and nurse autonomy [14,54]. Finally, pain management during other nursing and medical procedures (tracheal suctioning, central intravenous line placement...) was not evaluated. This should be a further step in our quality improvement project.

## Conclusions

A focused quality improvement project on pain management in the ICU was associated with improved pain management during patient turning for nursing procedures as determined by 1) a decreased incidence of severe pain; 2) an increased use of analgesic drugs; 3) a decreased incidence of serious adverse events. Careful documentation of pain management while moving ICU-patients for nursing procedures could be implemented as a health quality indicator in the ICU-setting.

## Key messages

• Moving an ICU patient for nursing care procedures is associated with serious adverse events in one out of three procedures.

• Serious adverse events are strongly associated with severe pain during these procedures.

• Health quality improvement of pain management is associated with a decrease of both severe pain and serious adverse events.

• Careful documentation of pain management while moving ICU patients could be implemented as a health quality indicator in the ICU setting.

## Abbreviations

BPS: Behavioral pain scale; BPS-NI: non-intubated BPS; COGAR: Comité d'Organisation et de Gestion de l'Anesthésie Réanimation du Centre Hospitalier Universitaire de Montpellier; ICU: Intensive care unit; NRS: Numeric rating scale; RASS: Richmond Agitation Sedation Scale; RN: Registered nurse; SAE: Serious adverse event; SAPS: Simplified Acute Physiological Score; WHO: World Health Organization

## Competing interests

The authors declare that they have no competing interests.

## Authors' contributions

GC, SJ, SDL, CG and MPS conceived the study and participated in its design and coordination. ADJ and NM performed the statistical analysis. ADJ, GC and SJ drafted the manuscript. MC and BJ helped to correct the manuscript. JC was in charge of English editing. All authors read and approved the final manuscript.

## Supplementary Material

Additional file 1 **Analgesia protocol for procedures - English language**. Poster referring to procedural pain management, created by the work group to highlight educational objectives and posted in every patient's room. English translation. Click here for file

Additional file 2 **Algorithm for continuous sedation-analgesia - English language**. Poster referring to continuous sedation-analgesia algorithm, adapted from [18] by the work group to highlight educational objectives and posted in every patient's room. English translation. Click here for file

Additional file 3 **Analgesia protocol for procedures - French language**. Poster referring to procedural pain management, created by the work group to highlight educational objectives and posted in every patient's room. Original French version. Click here for file

Additional file 4 **Algorithm for continuous sedation-analgesia - French language**. Poster referring to continuous sedation-analgesia algorithm, adapted from [18] by the work group to highlight educational objectives and posted in every patient's room. Original French version. Click here for file

Additional file 5 **Additional tables**. Table S1: Incidence of pain calculated on overall procedures for each of the four studied phases. Table S2: Sensitivity analysis of factors associated with serious adverse events determined by multivariate mixed-effects model analysis after removing tachycardia and/or hypertension from serious adverse events. Click here for file
