# Supplementary material for: Decreasing severe pain and serious adverse events while moving intensive care unit patients: a prospective interventional study (the NURSE-DO project)
Source: Crit Care. 2013 Apr 18;17(2):R74. doi: 10.1186/cc12683 (PMC3672726; doi:10.1186/cc12683)
Supplement: Additional file 1 — Analgesia protocol for procedures - English language. Poster referring to procedural pain management, created by the work group to highlight educational objectives and posted in every patient's room. English translation. [file cc12683-S1.PDF]

ANALGESIA PROCEDURE FOR NURSING CARE

CHRU MONTPELLIER
CENTRE HOSPITALIER REGIONAL UNIVERSITAIRE

NURSING (mobilization, drain removal...) = RISK = PATIENT MONITORING

CONTINUOUS SEDATION ANALGESIA YES

IS PATIENT PAINFUL AT REST ?

YES

Refer to continuous sedation-analgesia algorithm titrate sufentanil according to BPS score

until BPS <5 at rest

WAIT
for BPS at rest<5
before any nursing procedure

NO

Cf nurse report for the previous NURSING procedure
(Patient's Clinical-Information-System : data sheet (RASS, BPS/BPS-NI, V-NRS) and nurse shift reports (pain targets)

If it was not sufficient

according to sedation-analgesia algorithm :

* ↑sufentanil bolus of 5 µg

CONTINUOUS SEDATION ANALGESIA NO

IS PATIENT PAINFUL AT REST ?

YES

PAIN ALREADY KNOWN AND ITS CAUSE IS DIAGNOSED

YES

1) Registered nurses 'and nurse assistants' field of competence
(psychological support, bed installation, distraction, television-radio))

2) Non pharmacological therapies
(massage, music-thérapy)

3) Pharmacological therapies ordered for nursing procedures

NO

CALL DOCTOR

Alarm symptom : diagnosis is required

WAIT
for BPS/BPS-NI at rest<5
V-NRS<4
before any nursing procedure

NO

Cf nurse report of the previous nursing procedure
(Patient's Clinical-Information-System : data sheet (RASS, BPS/BPS-NI, V-NRS) and nurse shift reports (pain targets)

If not sufficient :

1. Reinforce
Non pharmacological therapies

2. Increase
Pharmacological therapies ordered for nursing procedures
***Acetaminophen** 1g/30 min
and/or
***Nefopam** 20 mg/30min,
and/or
***Tramadol** 50-100 mg/30 min
(peak action 30 min)

If ineffective :
***Alfentanil**
bolus of 0,25 à 0,5 mg/ 2min,
renew until 1mg IVD
(peak action :1-2 min)

FILL OUT Patient's Clinical-Information-System
1) At rest and during nursing procedure : RASS, V-NRS or BPS/BPS-NI
2) Pain targets (nurse shift reports)
- **a : type of nursing procedure + V-NRS/BPS/BPS-NI evaluated during the procedure**
- **b : analgesia provided**
- **c : efficacy /NRS-BPS/BPS-NI, incidents**

Working group :
Sedation-analgesia

CHRU de Montpellier - Direction de la Communication
et des Relations Publiques - 04 67 33 93 43 - G. Faugier - 08/10
