# Supplementary material for: Decreasing severe pain and serious adverse events while moving intensive care unit patients: a prospective interventional study (the NURSE-DO project)
Source: Crit Care. 2013 Apr 18;17(2):R74. doi: 10.1186/cc12683 (PMC3672726; doi:10.1186/cc12683)
Supplement: Additional file 2 — Algorithm for continuous sedation-analgesia - English language. Poster referring to continuous sedation-analgesia algorithm, adapted from [18] by the work group to highlight educational objectives and posted in every patient's room. English translation. [file cc12683-S2.PDF]

CHRU MONTPELLIER
CENTRE HOSPITALIER REGIONAL UNIVERSITAIRE

SEDATION PROTOCOL – SAINT ELOI HOSPITAL's ICU

REFERENCE MARK FOR MIND :

RASS ⟷ midazolam or propofol versus **BPS** ⟷ Sufentanil

Priority of BPS upon RASS(⟷ Sufentanil firstly if necessary)

CARE = bolus only, *never change the speed of continuous infusion*

versus **REST = modification of continuous infusion** ± titration (bolus)

Decrease both **sedative and Sufentanil** if overdose because **both are synergistic**!

Sufentanil = 5 µg/ml ; midazolam = 1mg/ml ; propofol = 10 mg/ml

AT REST

1) Firstly : BPS ≥5 whatever the RASS value

↓

*Sufentanil : titration, bolus of 1 ml/2 mn until BPS 3-4 (max 10 ml)

then ↑ **Sufentanil** of 1 ml/h (alert doctor if >10ml/h)

2) BPS 3-4 associated with a RASS value

RASS -5 or < target value

*↓ mdz or ppf of 1ml/h

*AND ↓ Sufentanil of 1ml/h

target value

no change

above target value

* mdz or ppf titration, bolus 1ml/2mn (max 10 ml) until RASS = target

* then ↑ mdz or ppf of 1 ml/h

(alert doctor if mdz>10ml/h or ppf >30 ml/h)

Titration and ↑ speed of continuous infusion: possible every hour

Decrease to reach the minimal efficient dose : every 4 hours [06h-12h-16h-20h-00h-04h]

≠DURING A CARE PROCEDURE : if BPS ≥ 5

Administer a bolus of **Sufentanil at least 5 mn before the procedure:**

*if 0≤SCI≤ 3ml/h : bolus of 1ml

*if 4≤ SCI≤6 ml/h : bolus of 2 ml

*if SCI>6ml/h : bolus of 3 ml

(SCI = speed of continuous infusion)

If the first dose was inefficient, increase by 1 ml the next time

AT ANY TIME if RASS ≥ 3 : mdz or ppf : 5ml, then alert the doctor

Revisibility index : 4 - 07 06 2010

Dr. Gerald CHANQUES

CHRU de Montpellier - Direction des Affaires Générales et Communication - 04 67 33 93 43 - G. Faugier - 09/12
