# Supplementary material for: Decreasing severe pain and serious adverse events while moving intensive care unit patients: a prospective interventional study (the NURSE-DO project)
Source: Crit Care. 2013 Apr 18;17(2):R74. doi: 10.1186/cc12683 (PMC3672726; doi:10.1186/cc12683)
Supplement: Additional file 3 — Analgesia protocol for procedures - French language. Poster referring to procedural pain management, created by the work group to highlight educational objectives and posted in every patient's room. Original French version. [file cc12683-S3.PDF]

PROCEDURE D'ANALGESIE LORS DES SOINS

CHRU MONTPELLIER
CENTRE HOSPITALIER REGIONAL UNIVERSITAIRE

SOIN (nursing, abaltion drain..) = RISQUE = PATIENT SCOPE-MONITORE

PROTOCOLE SEDATION CONTINUE PRESCRIT

Sufentanil ou ultiva prescrit

PATIENT ALGIQUE AU REPOS ?

OUI →

Selon Protocole sédation

Sur le BPS
titrer le Sufentanil
ou
augmenter le débit d'ultiva

jusqu'à BPS <5 au repos

→ **ATTENDRE**
BPS repos<5
avant de réaliser le soin

NON →

Cf procédure analgésique mise en place lors du soin précédent
(ICIP feuille de surv et transmission ciblée)

Si était insuffisante :

Selon Protocole sédation
*↑bolus sufentanil de 5µg
ou
*x3 au lieu de 2 le débit d'ultiva
Selon protocole

PROTOCOLE ANALGESIQUE « UNIVERSEL »

PATIENT ALGIQUE AU REPOS ?

OUI →

DOULEUR CONNUE ET DIAGNOSTIQUEE

OUI →

1) Rôle propre IDE/AS
(relation d'aide, installation, distraction : télé-radio-CD)

2) Thérapeutiques non médicamenteuses spécifiques
(massage®, musicothérapie)

3) Thérapeutiques médicamenteuses *prescrites* 'sur condition' 'si douleur'

NON →

Appel Médecin

Diagnostic symptome alarme

ATTENDRE
BPS /BPS-NI repos<5
EVN<4
avant de réaliser le soin

NON →

Cf procédure analgésique mise en place lors du soin précédent
(ICIP feuille de surv et transmission ciblée)

Si etait insuffisante :

1. Renforcer
Traitements non médicamenteux

2. Escalader
*Thérapeutiques médicamenteuses **prescrites** « sur condition » : avant soin soit :*
***Paracétamol** 1g/30 min
et/ou
***Acupan** 20 mg/30min,
et/ou
***Contramal** 50-100 mg/30 min
(pic action 30 min)

Si inefficace:
***Rapifen**
1mg dans 10ml ;
bolus de 0,25 à 0,5 mg/ 2min, renouveler jusqu'à 1mg IVD
(pic action 1-2 min)

RENSEIGNER

1) Repos et Soin RASS, EVN ou BPS/BPS-NI sur ICIP
2) Transmission ciblée douleur nursing et autres soins
D : type de soin + EVN/BPS/BPS-NI
A : actions réalisées
R : efficacité /EVN-BPS/BPS-NI, Incidents

Dr. Gerald CHANQUES
M.P. SUSBIELLES
Groupe Travail Sédation-analgésie

CHRU de Montpellier - Direction de la Communication et des Relations Publiques - 04 67 33 93 43 - G. Faugier - 07/10
