# Supplementary material for: Decreasing severe pain and serious adverse events while moving intensive care unit patients: a prospective interventional study (the NURSE-DO project)
Source: Crit Care. 2013 Apr 18;17(2):R74. doi: 10.1186/cc12683 (PMC3672726; doi:10.1186/cc12683)
Supplement: Additional file 4 — Algorithm for continuous sedation-analgesia - French language. Poster referring to continuous sedation-analgesia algorithm, adapted from [18] by the work group to highlight educational objectives and posted in every patient's room. Original French version. [file cc12683-S4.PDF]

CHRU MONTPELLIER
CENTRE HOSPITALIER REGIONAL UNIVERSITAIRE

PROTOCOLE DE « SEDATION-ANALGESIE »

Le BPS l'emporte sur le RASS (⟷ d'abord le Sufentanil si nécessaire)
Sufentanil (5µg/ml) Midazolam(1mg/ml) Propofol (10mg/ml)

Débuter hypnotique et sufentanil vit 2ml/h

AU REPOS
+/-TITRATION
Toujours MODIFICATION VITESSE CONTINUE !!!

1) Si BPS ≥ 5 quelque soit le RASS

↓

*Titrer **sufentanil** : bolus de 1 ml/2 min jusqu'à BPS 3-4 (max 10 ml)
*ET ↑ **sufentanil** de 1 ml/h (allo médecin si >10ml/h)

2) Si BPS 3-4 avec RASS

RASS -5 ou inférieur à la cible
*↓ mdz ou ppf de 1ml/h
*ET ↓ sufentanil de 1ml/h

Ciblé
Pas de Changement

Supérieur à la cible
*Titrer mdz ou ppf bolus 1ml/2min (max 10 ml) jusqu'à RASS cible
* ET ↑ mdz ou ppf
(allo médecin si mdz>10ml/ ou ppf>30ml/h)

Titration et ↑ vitesses continues : possible toutes les heures
Diminution pour **rechercher la posologie minimale efficace /h, min/4h**
EVALUER AU MOINS /4H

AU COURS D'UN SOIN : si BPS ≥ 5
Que des BOLUS de SUFENTANIL !!!
5min avant les soins car pic d'action une fois dans le sang : 5 min

*si 0≤vit≤ 3ml/h	: bolus de 1ml	*Si 1ère dose insuffisante, augmenter de 1 ml au soin suivant*
*si 4≤ vit≤6 ml/h	: bolus de 2 ml	
*si vit>6ml/h	: bolus de 3 ml	

Si le patient était en ventilation spontanée avant le bolus de sufentanil et que la ventilation d'apnée s'est activée
Après le bolus (sécurité), penser à déverrouiller la ventilation d'apnée 30 min après l'injection
(sinon le patient reste en ventilation d'apnée ; c.a.d. en contrôlé)

A TOUT MOMENT si RASS ≥ 3 : mdz ou ppf : 5ml et avertir le médecin

Indice de révisibilité : 4 - 07-06-10

Groupe Travail
Sédation-analgésie

Dr. Gerald CHANQUES

M.P. SUSBIELLES

CHRU de Montpellier - Direction de la Communication et des Relations Publiques - 04 67 33 93 43 - G. Faugier - 08/10
