# Supplementary material for: Decreasing severe pain and serious adverse events while moving intensive care unit patients: a prospective interventional study (the NURSE-DO project)
Source: Crit Care. 2013 Apr 18;17(2):R74. doi: 10.1186/cc12683 (PMC3672726; doi:10.1186/cc12683)
Supplement: Additional file 5 — Additional tables. Table S1: Incidence of pain calculated on overall procedures for each of the four studied phases. Table S2: Sensitivity analysis of factors associated with serious adverse events determined by multivariate mixed-effects model analysis after removing tachycardia and/or hypertension from serious adverse events. [file cc12683-S5.DOC]

**ADDITIONAL FILE 5**

**TITLE: Additional Tables**

**TABLE S1 p. 2**

###### Incidence of pain calculated on overall procedures for each of the 4 studied phases

**TABLE S2 p. 3**

**Sensitivity analysis of factors associated with Serious Adverse Events determined by multivariate mixed-effects model analysis after removing tachycardia and/or hypertension from Serious Adverse Events**

**TABLE S1**

###### Incidence of pain

###### calculated on overall procedures for each of the 4 studied phases

|  | **Phase-1**  **(baseline)**  **n= 182** | **Phase-2**  **(intervention)**  **n= 129** | **Phase-3**  **(adjustment)**  **n= 170** | **Phase-4**  **(consolidation)**  **n= 149** | p  1-2 | p  1-3 | p  1-4 |
| --- | --- | --- | --- | --- | --- | --- | --- |
|  |  |  |  |  |  |  |  |
| **Pain at rest** |  |  |  |  |  |  |  |
| Pain, n (%) | 51 (28%) | 28 (22%) | 39 (23%) | 22 (15%) | 0.92 | 0.77 | 0.09 |
| Moderate pain, n (%) | 37 (20%) | 23 (18%) | 32 (19%) | 20 (13%) | 0.58 | 0.78 | 0.51 |
| Severe pain, n (%) | 14 (8%) | 5 (4%) | 7 (4%) | 2 (1%) | 0.26 | 0.26 | 0.03 |
|  |  |  |  |  |  |  |  |
| **Pain during moving** |  |  |  |  |  |  |  |
| Pain, n (%) | 81 (44%) | 45 (35%) | 59 (34%) | 35 (23%) | 0.27 | 0.32 | 0.05 |
| Moderate pain, n (%) | 51 (28%) | 33 (26%) | 48 (28%) | 32 (21%) | 0.90 | 0.67 | 0.46 |
| Severe pain, n (%) | 30 (16%) | 12 (9%) | 11 (6%) | 3 (2%) | 0.22 | 0.04 | 0.02 |

Statistical analysis was performed using a generalized linear mixed-effects model for repeated measures.

**TABLE S2**

**Sensitivity analysis of factors associated with Serious Adverse Events**

**determined by multivariate mixed-effects model analysis**

**after removing tachycardia and/or hypertension from Serious Adverse Events**

A. All Serious Adverse Events included except tachycardia

(i.e. cardiac arrest, arrhythmias, bradycardia, hypertension,

hypotension, desaturation, bradypnea or ventilatory distress)

|  | **OR**  **[95% CI]** | **p** |
| --- | --- | --- |
|  |  |  |
| Phase-3, n (%) | 0.45 [0.24;0.81] | < 0.01 |
| Phase-4, n (%) | 0.52 [0.29;0.93] | 0.03 |
| Intubation status, n (%) | 2.17 [1.44;3.27] | < 0.001 |
| Severe pain during moving, n (%) | 2.69 [1.50;4.83] | < 0.001 |

B. All Serious Adverse Events included except hypertension

(i.e. cardiac arrest, arrhythmias, tachycardia, bradycardia,

hypotension, desaturation, bradypnea or ventilatory distress)

|  | **OR**  **[95% CI]** | **p** |
| --- | --- | --- |
|  |  |  |
| Phase-3, n (%) | 0.47 [0.26;0.85] | 0.01 |
| Phase-4, n (%) | 0.43 [0.23;0.79] | < 0.01 |
| Intubation status, n (%) | 2.25 [1.49;3.39] | < 0.001 |
| Severe pain during moving, n (%) | 3.01 [1.69;5.39] | < 0.001 |

C. All Serious Adverse Events included except tachycardia and hypertension

(i.e. cardiac arrest, arrhythmias, bradycardia, hypotension,

desaturation, bradypnea or ventilatory distress)

|  | **OR**  **[95% CI]** | **p** |
| --- | --- | --- |
|  |  |  |
| Phase-3, n (%) | 0.51 [0.27;0.97] | 0.04 |
| Phase-4, n (%) | 0.39 [0.20;0.77] | < 0.01 |
| Intubation status, n (%) | 2.64 [1.70;4.08] | < 0.001 |
| Severe pain during moving, n (%) | 3.14 [1.72;5.72] | < 0.001 |
